# Elevated Carbon Dioxide Altered Morphological and Anatomical Characteristics, Ascorbic Acid Accumulation, and Related Gene Expression during Taproot Development in Carrots

**DOI:** 10.3389/fpls.2016.02026

**Published:** 2017-01-05

**Authors:** Xue-Jun Wu, Sheng Sun, Guo-Ming Xing, Guang-Long Wang, Feng Wang, Zhi-Sheng Xu, Yong-Sheng Tian, Xi-Lin Hou, Ai-Sheng Xiong

**Affiliations:** ^1^State Key Laboratory of Crop Genetics and Germplasm Enhancement, College of Horticulture, Nanjing Agricultural UniversityNanjing, China; ^2^College of Horticulture, Shanxi Agricultural UniversityTaigu, China

**Keywords:** elevated CO_2_, ascorbic acid, anatomical structure, transcript levels, taproot, *Daucus carota* L.

## Abstract

The CO_2_ concentration in the atmosphere has increased significantly in recent decades and is projected to rise in the future. The effects of elevated CO_2_ concentrations on morphological and anatomical characteristics, and nutrient accumulation have been determined in several plant species. Carrot is an important vegetable and the effects of elevated CO_2_ on carrots remain unclear. To investigate the effects of elevated CO_2_ on the growth of carrots, two carrot cultivars (‘Kurodagosun’ and ‘Deep purple’) were treated with ambient CO_2_ (a[CO_2_], 400 μmol⋅mol^-1^) and elevated CO_2_ (e[CO_2_], 3000 μmol⋅mol^-1^) concentrations. Under e[CO_2_] conditions, taproot and shoot fresh weights and the root/shoot ratio of carrot significantly decreased as compared with the control group. Elevated CO_2_ resulted in obvious changes in anatomy and ascorbic acid accumulation in carrot roots. Moreover, the transcript profiles of 12 genes related to AsA biosynthesis and recycling were altered in response to e[CO_2_]. The ‘Kurodagosun’ and ‘Deep purple’ carrots differed in sensitivity to e[CO_2_]. The inhibited carrot taproot and shoot growth treated with e[CO_2_] could partly lead to changes in xylem development. This study provided novel insights into the effects of e[CO_2_] on the growth and development of carrots.

## Introduction

According to the reports of the America’s National Oceanic and Atmospheric Administration (NOAA), the concentration of CO_2_ in the atmosphere has increased from 280 μmol⋅mol^-1^ in pre-industrial times to 400 μmol⋅mol^-1^ at present and is continuing to rise in the future (Leakey et al., 2009). The elevated atmospheric CO_2_ concentration is a major component of global climate change. CO_2_ is an essential substrate for plant photosynthesis. Over the last two decades, the positive impacts of elevated CO_2_ on plants have been detected (Urban, 2003), including increased photosynthetic rate, enhanced photosynthate accumulation. In the plant production, the CO_2_ has been used to enhance vegetative growth, increase crop yield, and improve crop quality (Bugbee et al., 1994; Kauder et al., 2000). Although the physiological responses under excess concentration of CO_2_ (>1200 μmol⋅mol^-1^) were different from that under a concentration of CO_2_ (400–1200 μmol⋅mol^-1^; Kaplan et al., 2012), the 3000 and 4000 μmol⋅mol^-1^ concentrations have been used as in e[CO_2_] treatment in some plants (Wang M. J. et al., 2015).

In wheat (2600 μmol⋅mol^-1^ CO_2_; Reuveni and Bugbee, 1997) and bean plant (5000 μmol⋅mol^-1^ CO_2_; Wheeler et al., 1993; Jolliffe and Ehret, 2011), the higher CO_2_ enrichment results in decreased yield. The stomatal conductance has no apparent inhibition under 1000 μmol⋅mol^-1^ CO_2_ but gradually decreased in the presence of 3000 μmol⋅mol^-1^ CO_2_ for both C_3_ soybean and C_4_ maize (Wang M. J. et al., 2015). Under 3000 μmol⋅mol^-1^ CO_2_, the carboxylation efficiency was declined than that under 1000 μmol⋅mol^-1^ CO_2_ in soybean and maize. The e[CO_2_] also induced necrosis and chlorosis of leaves. In addition, plants show some morphological and physiological changes under e[CO_2_] concentration (Mamatha et al., 2014). Under e[CO_2_], a steep CO_2_ concentration gradient was observed between the outside and inside of the leaf, allowing great amounts of CO_2_ to diffuse into the leaf (Singh and Agrawal, 2015). In that case, the increased CO_2_/O_2_ ratio at the sites of photo reduction can reduce the rate of oxygen activation and ROS formation (Gutteridge and Halliwell, 1992).

Elevated CO_2_ may also have an effect on antioxidant substances. Plants possess non-enzymatic antioxidant systems, which help prevent oxidative damage and maintain cellular homeostasis (Hussain et al., 2011). The non-enzymatic antioxidant system is composed of several low-molecular-weight antioxidant molecules, such as AsA, which can directly eliminate ROS and regenerate ROS detoxifying enzymatic cooperative systems (Karahalil et al., 2015). AsA is a water-soluble antioxidant vitamin and a ubiquitous nutrient in eukaryotes (Bozonet et al., 2015; Flores-Félix et al., 2015). Fruits and vegetables are the major sources of ascorbate (Cruz-Rus et al., 2011; Ren et al., 2013). AsA is also involved in the regulation of photosynthesis and electron transport in the membranes (Chatterjee, 1973; Horemans et al., 1994; Ivanov, 2014). At present, almost all the steps of four AsA *de novo* synthesis pathways have been revealed in the past decades (Smirnoff et al., 2001; Wolucka and Van Montagu, 2007; Alos et al., 2013). The AsA synthesis pathways were the L-galactose, L-gulose, *myo*-inositol, and D-galacturonic acid pathways (Wang G. L. et al., 2015).

Carrot (*Daucus carota* L.), a member of the Apiaceae, is an important vegetable crop worldwide because of its edible root and excellent source of vitamins and fibers in the diet. Carrot is a biannual diploid (2*n* = 2*x* = 18) with AsA, which is an important material for antioxidant capacity. Compared with the broccoli (3.25–7.64 mg g^-1^ FW), radish (1.64-3.03 mg g^-1^ FW) (Xu et al., 2013) and blueberry (0.03–0.08 mg g^-1^ FW), the AsA levels in ‘Kurodagosun’ is under the range of 0.127–0.163 mg g^-1^ FW (Wang G. L. et al., 2015). Besides, little is known about the molecular responses to elevated CO_2_ in carrot. In this study, our objective was to investigate the effects of elevated carbon dioxide on carrot morphological and anatomical characteristics, AsA accumulation, and expression profiles of AsA-related genes in two carrot cultivars (cvs. ‘Kurodagosun’ and ‘Deep purple’).

## Materials and Methods

### Carrot Growth Conditions and Treatments

‘Kurodagosun’ and ‘Deep purple’ are commonly cultivated carrot cultivars and were selected for analysis in this work (**Supplementary Figure S1**). ‘Kurodagosun’ is an early maturing variety with an orange root and high resistance against abiotic stress. ‘Deep purple’ is a late maturing variety with a purple and conical root. The seeds of the two carrot cultivars were sown in a chamber at the State Key Laboratory Genetics and Germplasm Enhancement in Nanjing Agricultural University, Jiangsu Province, China (32°02′N, 118°50′E). The chamber condition was programmed for a relative humidity of 60–70% with a photoperiod of 16 h light (300 μ mol m^-2^ s^-1^) and 8 h dark at day/night temperature of 25/18°C. The plants were grown under normal conditions (387 μmol⋅mol^-1^ CO_2_ and 21% oxygen in the atmosphere) for 38 days. Afterward, carrot plants were divided into two groups and transferred to two chambers with similar growth conditions. Simultaneously, the plants were subjected to 400 or 3000 μmol⋅mol^-1^ CO_2_, respectively, for another 30 days. To investigate AsA accumulation and expression profiles of AsA-related genes, carrot root samples from the two cultivars were harvested at 10 am at 38, 48, 58, and 68 DAS. Each sample was immediately frozen in liquid nitrogen and stored at -70°C until RNA isolation. Three independent replicates were used for each treatment.

## Morphological and Anatomical Analysis

**FIGURE 1:**
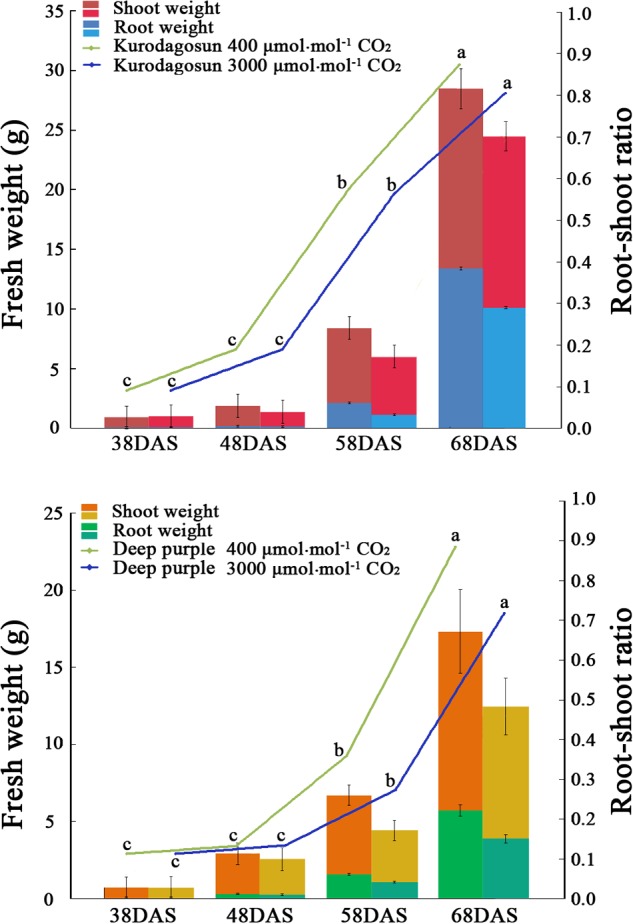
**Effects of a[CO_2_] and e[CO_2_] on carrot fresh weight and root/shoot ratio.** Error bars represent the standard deviation (SD) in three independent replicates. Different letters indicate statistically significant differences (*P* < 0.05).

The carrots were sampled at 38, 48, 58, and 68 DAS for analysis of the root and shoot biomass accumulation. Carrot roots and shoots were measured from three randomly selected plants per replicate, and the values were derived from the average of three replicate measurements. An orange and purple color first appeared on the taproot surface of ‘Kurodagosun’ and ‘Deep purple’ at 38 DAS, respectively (**Supplementary Figures S1** and **S2**). The color of the roots gradually became darker as the roots enlarged. To characterize carrot growth and development, the root/shoot ratio was measured in carrots during different developmental stages (**Figure 1**). Fresh samples of ‘Kurodagosun’ and ‘Deep purple’ were also harvested for anatomical structure analysis. Samples were cut into about 2 mm-thick slices and then immediately stored in phosphate buffer (pH 7.2) with 2.5% glutaraldehyde solution. Subsequently, the samples were used for safranin O/fast green staining. First, the sheet was dewaxed in xylene for 25 min and dehydrated with different concentrations of ethanol solution. Second, the samples were stained with 1% safranin O for 2 h and dehydrated in a series of ethanol with different concentrations. Afterward, the samples were counterstained with 0.5% green for 15 s, and excess stain was washed out using 95% ethanol and absolute ethanol. The lignin and fiber tissues were stained with red and green, respectively, and observed under a light microscope.

### AsA Content Analysis

Determination of AsA levels was performed using the 4, 7-diphenyl-1, 10-phenanthroline method described by Arakawa et al. (1981). In brief, 0.8 g of fresh samples was pulverized with 2 mL of 0.3 M TCA in a mortar. The extract was centrifuged at 12,000 *g* for 15 min to obtain 0.5 mL of supernatant. Subsequently, 1.5 mL of 0.3 M TCA, 1 mL of EtOH, 0.5 mL of 0.4% (v/v) H_3_PO_4_-EtOH, 1 mL of 0.5% (w/v) BP-EtOH, and 0.5 mL of 0.03% (w/v) FeCl_3_-EtOH were added in order. The mixture was incubated at 30°C for 90 min, and absorbance was measured at 534 nm. To measure the T-AsA levels, 0.5 mL of 60 mM dithiothreitol was added into 0.5 mL of supernatant, and 0.2 M Na_2_HPO_4_–1.2 M NaOH was introduced to adjust the pH to 7.0. After reaction for 10 min, the pH was adjusted to 1.5 using 0.3 M TCA. Calibration curves were constructed for both AsA and T-AsA.

### RNA Extraction and cDNA Synthesis

Carrot samples from different developmental stages and treatments were ground in liquid nitrogen. The total RNA kit (Tiangen, Beijing, China) was used to extract total RNA according to the manufacturer’s instructions. About 10 μg of RNA was used to synthesize first-strand cDNA using the Prime Script RT reagent kit (TaKaRa, Dalian, China). The cDNA was diluted 18 times for PCR amplification.

### Quantitative Real-Time PCR (qPCR) Analysis

Based on the homologous sequences from *Arabidopsis* and other plant species, a total of 12 genes involved in AsA biosynthesis, degradation, and recycling were identified in CarrotDB (Xu et al., 2014; Wang G. L. et al., 2015). These genes included phosphoglucose isomerase (*DcPGI*), phosphomannose isomerase (*DcPMI*), GDP-D-manmose pyrophosphorylase (*DcGMP*), GDP-D-mannose-3′,5′-epimerase (*DcGME*), GDP-L-galactose phosphorylase (*DcGGP*), L-galactose-1-P phosphatase (*DcGPP*), *myo*-inositol oxygenase (*DcMIOX*), ascorbate oxidase (*DcAO*), ascorbic acid peroxidase (*DcAPX*), monodehydroascorbate reductase (*DcMDHAR*), dehydroascorbate reductase (*DcDHAR*), and glutathione reductase (*DcGR*). A total of six genes (*DcPGI*, *DcPMI*, *DcGMP*, *DcGME*, *DcGGP*, and *DcGPP*) were identified in the L-galactose pathway. *DcMIOX* were involved in the *myo*-inositol and D-galacturonic acid pathways, respectively. We have submitted the nucleotide sequences to GenBank, and the corresponding accession numbers were KY347803 (*DcGGP*), KY347804 (*DcGME*), KY347805 (*DcGMP*), KY347806 (*DcGPP*), KY347807 (*DcPMI*), KR364573.1 (*DcAPX*), KY347808 (*DcPGI*), KY347809 (*DcAO*), KY347810 (*DcDHAR*), KY347811 (*DcGR*), KY347812 (*DcMDHAR*), KY347813 (*DcMIOX*) (**Supplementary Table S2**).

Quantitative Real-Time PCR analysis was conducted with an SYBR Premix *Ex Taq* kit, and the primers used for qPCR were designed with Primer 6.0 software (**Supplementary Table S1**). The data were analyzed by iQ5 software and the iQ5 Real-time PCR System according to the manufacturer’s instruction as follows: 95°C for 30 s, followed by 40 cycles at 95°C for 5 s, 60°C for 30 s, and melting curve analysis. The *tubulin* gene has been identified as the suitable reference genes for the normalization of gene expression in carrot at different developmental stages (Wang et al., 2016) and under the abiotic stresses (Tian et al., 2015). The *tubulin* gene of carrot was chosen to normalize the expression levels of the AsA biosynthesis and recycling genes in two carrot cultivars under two carbon dioxide concentration treatments. The reaction system contained 10 μL of SYBR Premix *Ex Taq*, 7.2 μL of deionized water, 0.4 μL of each primer, and 2 μL of diluted cDNA. 2^-ΔΔCT^ method was used to measure the RNA level, which were expressed relative to the *tubulin* gene (Pfaffl, 2001). The values for the mean expression and standard deviation (SD) were calculated from the results of three independent biological replicates.

### Statistical Analysis

Statistical analyses were performed using SPSS16.0 software (Shi et al., 2013). Mean values ±SD of three replicates were recorded. The significance of elevated carbon dioxide concentration effects compared with controls was assessed in a two-way analysis of variance (ANOVA). Significant differences were defined as *P* < 0.05 in Tukey’s post-test.

## Results

### Growth Analysis of Carrot Roots in Two Cultivars

Different morphological characteristics were identified in ‘Kurodagosun’ and ‘Deep purple’ under a[CO_2_] and e[CO_2_] treatments (**Supplementary Figures S1** and **S2**). Under the normal condition, the fresh weights of shoots were 15.07 g and 12.27 g at 68 DAS in ‘Kurodagosun’ and ‘Deep purple,’ respectively. Similarly, the fresh weights of roots were 13.40 g and 5.71 g at 68 DAS in ‘Kurodagosun’ and ‘Deep purple,’ respectively. During the carrot development, the fresh weight of shoots and roots were most increased at 58 to 68 DAS. The fresh weights of roots and shoots under a[CO_2_] treatment were also much heavier than those under elevated CO_2_ treatment both of two carrot cultivars. In the presence of e[CO_2_], the shoot biomass showed a 4.31 and 28.3% decrease in ‘Kurodagosun’ and ‘Deep purple’ at 68 DAS, respectively, when compared to plants grown under a[CO_2_]. The root biomass also decreased by 24.4 and 34.0% in the two cultivars due to e[CO_2_] at 68 DAS. The root/shoot ratio per cultivar of the a[CO_2_] and e[CO_2_] treatments increased slightly over the course of the experiment. Significant changes of the root/shoot ratio within cultivars were found at 58 and 68 DAS (**Figure 1**).

### Anatomical Structure Analysis of Carrot Taproots under a[CO_2_] and e[CO_2_] Treatments

**FIGURE 2:**
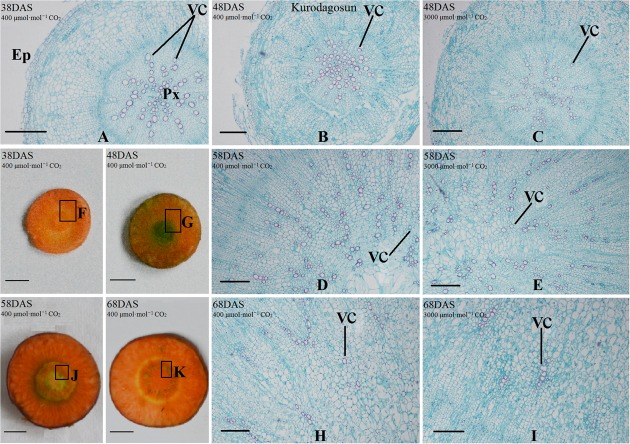
**Safranin O/fast green staining on the carrot taproot anatomical structure of ‘Kurodagosun.’** Px, protoxylem; VC, vascular cambium; PP, primary phloem; Ep, epidermis. Scale bar in **(A)** is 50 μm; scale bars in **(B–K)** are 40 μm.

**FIGURE 3:**
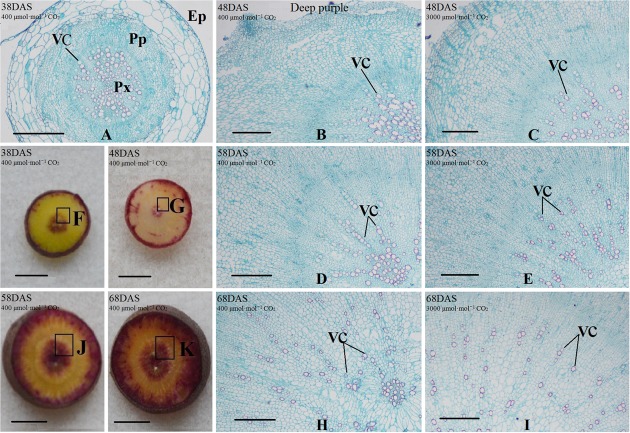
**Safranin O/fast green staining on the carrot taproot anatomical structure of ‘Deep purple.’** Px, protoxylem; VC, vascular cambium; PP, primary phloem; Ep, epidermis. Scale bar in **(A)** is 50 μm; scale bars in **(B–K)** are 40 μm.

Safranin-O/fast green staining was used to investigate the anatomical structure of carrot taproot tissues containing lignified cell walls that stain brilliant red. Px, VC, PP, and epidermis were observed at 38 DAS (**Figures 2** and **3**). Under normal conditions, the size of VC located between Px and PP also increased during ‘Kurdogosun’ growth. The carrot roots thickened along with the development of the Px, PP, and VC. The parenchymal cells became larger, and the roots continued to expand. In the presence of e[CO_2_], the increased size of VC and lager parenchymal cells were also showed from 48 DAS to 68 DAS in ‘Kurdogosun.’ In addition, the ratio of xylem area to total root area were decreased than that in a[CO_2_] of ‘Kurdogosun.’ Anatomical structure in ‘Deep purple’ was similar to that in ‘Kurdogosun’ of both a[CO_2_] and e[CO_2_] treatments.

### AsA and T-AsA Levels in Carrot Taproots under a[CO_2_] and e[CO_2_] Treatments

**FIGURE 4:**
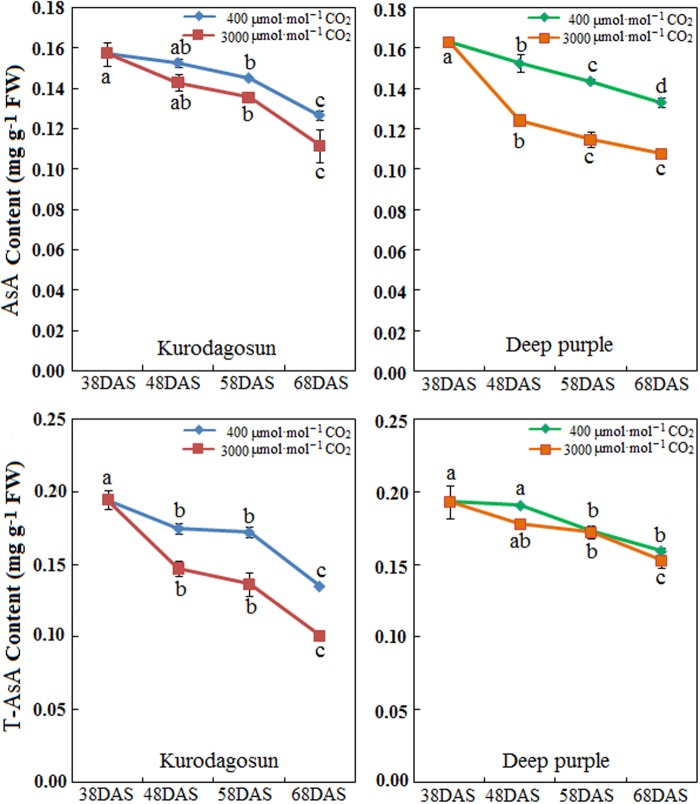
**Levels of AsA and T-AsA in ‘Kurodagosun’ and ‘Deep purple’ of the carrot taproot under a[CO_2_] and e[CO_2_].** Error bars represent SD in three independent replicates. Different letters in columns indicate statistically significant differences (*P* < 0.05).

Carrot taproots of ‘Kurodagosun’ and ‘Deep purple’ at different developmental stages were used to investigate AsA and T-AsA contents (**Figure 4**). The AsA contents of both cultivars peaked at 38 DAS and then decreased gradually from 48 to 68 DAS. Compared with the control plants, the levels of AsA and T-AsA under e[CO_2_] slightly decreased in the two carrot cultivars. ‘Deep purple’ and ‘Kurodagosun’ had the highest AsA levels of 0.163 mg⋅g^-1^ FW and 0.157 mg⋅g^-1^ FW, respectively. The highest T-ASA values in ‘Kurodagosun’ and ‘Deep purple’ were about 0.195 mg⋅g^-1^ FW and 0.193 mg⋅g^-1^ FW, respectively.

### Expression Profiles of AsA Biosynthetic Genes in ‘Kurodagosun’ Taproot

**FIGURE 5:**
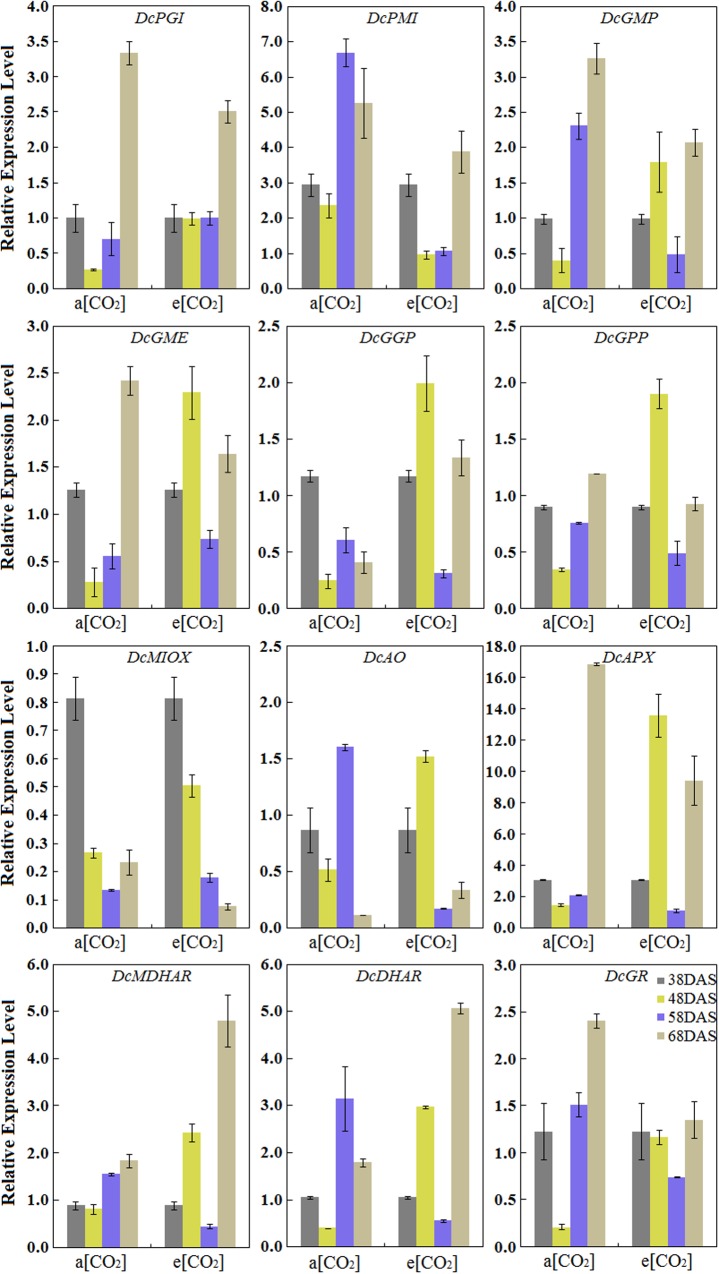
**Transcript profiles of AsA biosynthetic pathways in ‘Kurodagosun.’** a[CO_2_] and e[CO_2_] are represent 400 and 3000 μmol⋅mol^-1^ CO_2_ concentration. Error bars represent the SD in three independent replicates.

The results showed that the eight genes involved in the L-galactose pathway exhibited relatively higher expression levels than the genes in the other biosynthetic pathways. Under normal conditions, the pattern of transcript levels of *DcPGI*, *DcGMP*, *DcGME*, *DcGPP*, *DcAPX*, *DcMDHAR*, and *DcGR* have closely resembled patterns and reached the highest level at 68 DAS. The genes of *DcPMI*, *DcAO*, and *DcDHAR* were highly expressed at the 58 DAS, and then decreased at the last stage. *DcGGP* and *DcMIOX* were highly expressed at 38 DAS. The results also demonstrated the effects on gene expression levels at a[CO_2_] and e[CO_2_] concentrations. In the presence of elevated CO_2_, the relative expression levels of some genes, such as *DcGME*, *DcGGP*, *DcGPP*, *DcAO*, and *DcAPX* exhibited similar patterns and peaked at 48 DAS, which decreased at 58 DAS, increased again at the last stage. The expression levels of *DcMIOX* decreased with carrot growth, and these levels were strongly consistent with AsA contents (**Figure 5**).

### Expression Profiles of AsA Biosynthetic Genes in ‘Deep purple’ Taproot

**FIGURE 6:**
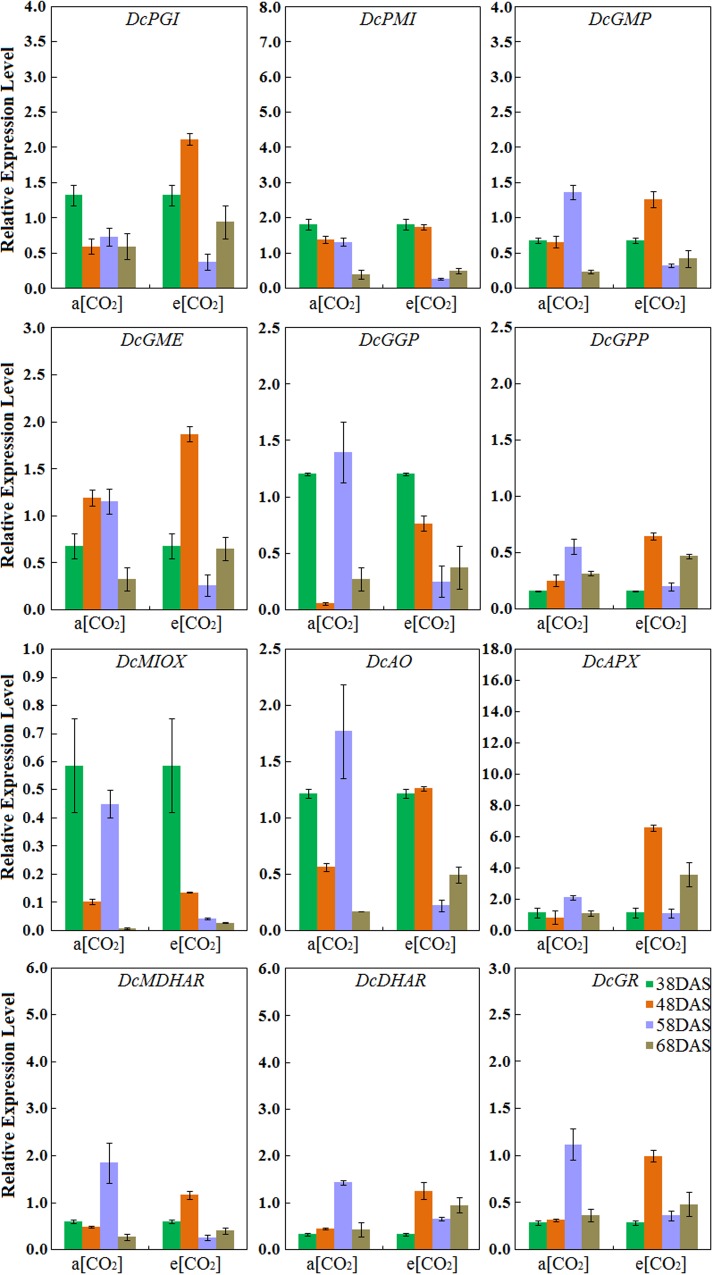
**Transcript profiles of AsA biosynthetic pathways in ‘Deep purple.’** a[CO_2_] and e[CO_2_] are represent 400 and 3000 μmol⋅mol^-1^ CO_2_ concentration. Error bars represent the SD in three independent replicates.

Under normal conditions, the expression levels of *DcPMI* peaked at 38 DAS and gradually decreased during root development, which was consistent with AsA accumulation. The expression patterns of *DcGMP*, *DcGGP*, *DcGPP*, *DcAO*, *DcAPX*, *DcMDHAR*, *DcDHAR*, and *DcGR* in ‘Deep purple’ were highest at 58 DAS. Among these genes, the transcript levels of *DcAO* and *DcDHAR* were similar to those in ‘Kurodagosun’ (**Figure 6**). Among these nine genes, in the presence of elevated CO_2_, the different expression levels were showed the five AsA degradation and recycling genes, *DcAO*, *DcAPX*, *DcMDHAR*, *DcDHAR*, and *DcGR*, were peaked at 48 DAS. The expression profiles of four genes, namely, *DcPGI*, *DcGMP*, and *DcGME* peaked at 48 DAS and showed the lowest expression levels at 58 DAS. The expression profile of *DcMIOX* was strongly consistent with the AsA accumulations during carrot development (**Figure 6**).

## Discussion

In the current study, a visual depiction showing the growth differences between the a[CO_2_] and e[CO_2_] treatments was provided in **Supplementary Figures S1** and **S2**. The root/shoot ratio of carrot under e[CO_2_] was lower than that under a[CO_2_], which was consistent with several studies reported previously (Schultz, 2000). This phenomenon suggested that the e[CO_2_] induced stomatal closure; therefore, the CO_2_ fixation may be also repressed. The changes in carrot morphological structure under high CO_2_ concentration were mainly induced by alterations in the CO_2_/O_2_ ratio. The taproot anatomical characteristics were also changed under the elevated CO_2_ concentration. Carrot root exhibited smaller xylem area under e[CO_2_], in accordance with the findings from a previous research (Richet et al., 2011). The newly synthesized xylem was different from constitutive xylem and was thought to be involved in defense mechanisms. Plant hormones such as auxin and cytokinin played roles in cell division, but whether changes in xylem development altered by e[CO_2_] is related to hormone accumulation remains unclear.

In the present study, the highest AsA content in carrot taproot was measured at 38 DAS and gradually decreased at the remaining DAS. Plants including apple (Bulley et al., 2009), peach (Imai et al., 2009), and kiwifruit (Li et al., 2010) also accumulated a high level of AsA during early fruit development. Under normal conditions, the content of AsA in carrot taproot was 0.127–0.163 mg g^-1^ FW in the two carrot cultivars. Previous studies have reported AsA accumulation during carrot root development (Wang G. L. et al., 2015), and the findings were consistent with our study. In plants, the AsA accumulation was reported to change in response to light (Tabata et al., 2002), times of day (Chen and Gallie, 2004), age (Bartoli et al., 2000), cell compartment (Zechmann et al., 2011), and carbon dioxide. Under concentration of CO_2_ (400–1200 μmol⋅mol^-1^), the consistency of increased content of ascorbic acid and enriched CO_2_ in plants of orange, leaf lettuce, and tomato were demonstrated (Zhang et al., 2014). Differed from the above, in this study, AsA levels in both ‘Kurodagosun’ and ‘Deep purple’ were lower under e[CO_2_] than that under normal conditions, which may resulted in a decrease in antioxidant defensive capacity under e[CO_2_] (>1200 μmol⋅mol^-1^). Further studies showed that the decreased ascorbate content may be attributed to low levels of photooxidative stress under e[CO_2_] (Ainsworth et al., 2008; Gillespie et al., 2011).

Some genes encoding enzymes in the AsA biosynthetic pathway may also play essential roles in other metabolic routes. For example, glucose-6-phosphate isomerase (GPI) is an important enzyme in AsA biosynthetic route, and it is also implicated in sugar metabolism (Cocetta et al., 2012). GME was identified as one of the key rate-limiting enzymes in the AsA biosynthetic pathway, but also played a role in the biosynthesis of cell wall polysaccharides (Gilbert et al., 2009). Moreover, the activity of GGP protein has been investigated as an important role in AsA biosynthesis and it was reported to be affected by light intensity. Therefore, the expression patterns of related genes may be not correlated well with AsA accumulation. The inconsistency between the transcript levels and AsA accumulation during carrot root development may also be a result of the combination of several regulatory mechanisms, such as pre-transcriptional and post-transcriptional mechanisms.

The AsA degradation and recycling pathways also played important roles in the response and adaptation to stress (Veltman et al., 1999). There are shown that AsA consumption and recycling is mainly determined by oxidation of AO and APX, and reduction of MDHAR, DHAR and GR. Both of the higher oxidizing activities and lower recycling activities could result in decreased AsA levels. Namely, compared with that in a[CO_2_], more transcripts of *DcAO* and *DcAPX* were detected at 48 DAS when the root suffered from e[CO_2_] in the two cultivars, whereas the AsA accumulation were decreased. Furthermore, GR also played a key role in other important mechanisms in plants, such as involvement in the cell’s scavenging system for reactive oxygen species by reducing glutathione disulfide (GSSG) to glutathione (GSH).

## Conclusion

The present study found that the root/shoot ratio in two carrot cultivars decreased in the presence of elevated CO_2_ (e[CO_2_], 3000 μmol⋅mol^-1^). Compared with plants grown under a[CO_2_], the proportion of xylem region was smaller under e[CO_2_] in carrot taproot of both cultivars. These results suggested that 3000 μmol⋅mol^-1^ CO_2_ inhibited carrot growth and development. The AsA levels decreased in carrots under elevated CO_2_ (e[CO_2_], 3000 μmol⋅mol^-1^) treatment. The transcript profiles of most genes (*DcPGI*, *DcPMI*, *DcGMP*, *DcGME*, *DcGGP*, *DcGPP*, *DcAO*, *DcAPX*, *DcMDHAR*, *DcDHAR*, and *DcGR*) were not well correlated with AsA accumulation under a[CO_2_] (400 μmol⋅mol^-1^) and e[CO_2_] (3000 μmol⋅mol^-1^) treatments. The results of the present work suggested that the AsA accumulation response to CO_2_ in carrot taproot resulted from a complex metabolic network, including biosynthesis, recycling, and degradation pathways. The AsA content may also differed in different genotypes of carrot under elevated CO_2_ treatment.

## Author Contributions

A-SX and X-JW conceived and designed the experiments. X-JW, G-LW, FW, Z-SX, Y-ST, and A-SX performed the experiments. X-JW, SS, G-MX, and A-SX analyzed the data. A-SX contributed reagents/materials/analysis tools. X-JW wrote the paper. A-SX, X-LH, and X-JW revised the paper.

## Conflict of Interest Statement

The authors declare that the research was conducted in the absence of any commercial or financial relationships that could be construed as a potential conflict of interest.

## Abbreviations

a[CO_2_]: ambient CO_2_
AO: ascorbate oxidase
APX: ascorbate peroxidase
AsA: L-Ascorbic acid
CO_2_: carbon dioxide
DAS: days after sowing
DHAR: dehydroascorbate reductase
e[CO_2_]: elevated CO_2_
Ep: epidermis
EtOH: ethyl alcohol
FW: fresh weight
GGP: GDP-L-galactose transferase
GME: GDP-mannose-3′,5′-epimerase
GMP: GDP-mannose pyrophosphorylase
GPP: L-galatose-1-phosphate phosphatase
HXK: hexokinase
MIOX: *myo*-inositol oxygenase
MDHAR: monodehydroascorbate reductase
PGI: phosphoglucose isomerase
PMI: phosphomannose isomerase
PP: primary phloem
Px: protoxylem
qPCR: quantitative real-time PCR
ROS: reactive oxygen species
T-AsA: total ascorbic acid (AsA + DHA)
TCA: trichloroacetic acid
VC: vascular cambium
