# Supplementary material for: Elevated Carbon Dioxide Altered Morphological and Anatomical Characteristics, Ascorbic Acid Accumulation, and Related Gene Expression during Taproot Development in Carrots
Source: Front Plant Sci. 2017 Jan 5;7:2026. doi: 10.3389/fpls.2016.02026 (PMC5221676; doi:10.3389/fpls.2016.02026)

**Elevated carbon dioxide altered morphological and anatomical characteristics, ascorbi****c acid accumulation, and related gene expression during taproot development in carrots**

Xue-Jun Wu ^1^, Sheng Sun ^2^, Guo-Ming Xing ^2^, Guang-Long Wang ^1^, Feng Wang ^1^, Zhi-Sheng Xu ^1^, Yong-Sheng Tian ^1^, Ai-Sheng Xiong ^1,*^

1. State Key Laboratory of Crop Genetics and Germplasm Enhancement, College of Horticulture, Nanjing Agricultural University, Nanjing, China

2. College of Horticulture, Shanxi Agricultural University, Taigu, China

* Please address all correspondence to: A.S. Xiong ([xiongaisheng@njau.edu.cn](mailto:xiongaisheng@njau.edu.cn))

---------------

Dr. Ai-Sheng Xiong

Professor

State Key Laboratory of Crop Genetics and Germplasm Enhancement,

College of Horticulture,

Nanjing Agricultural University,

Nanjing, 210095, China

Email: [xiongaisheng@njau.edu.cn](mailto:Xiongaisheng@njau.edu.cn)

***Running title:*** *Effect of CO_2_ on AsA accumulation in carrot*

**Supplementary Data**

**Table S1** Primer sequences used for RT-PCR of related genes of ascorbic acid biosynthesis and recycling pathways in carrot taproot.

| Gene name | Forward primer  (5'–3') | Reverse primer  (5'–3') | Function |
| --- | --- | --- | --- |
| *DcPGI* | GTGCCACTACTGACACTATTGATAAGC | TCTGTGCTGTTAATACGCTCTCCATT | biosynthesis |
| *DcPMI* | GTCACAGCAGGCAATGGAACAATG | CCAGCTCGGTACAGATGCAACTC | biosynthesis |
| *DcGMP* | CCTTATTCCGTTGGAGGGTTTGGA | AAGAAGTTGAGCACCTCTGGTT | biosynthesis |
| *DcGME* | CATACACCTACGAGAGGCCTTGAGAG | TCCAGTCAGAAGCAATGATGTAATGTC | biosynthesis |
| *DcGGP* | CGCTACGATGTTACTGCCTGTGA | CACCTCTTCCTGCCCAACTTTAGT | biosynthesis |
| *DcGPP* | AACTGCTGCCAAGACTGGTTCTG | ATGAATCTCCTATAAGCCCTCCTCCT | biosynthesis |
| *DcMIOX* | GCATCATGGAGGAGCGTACAGAC | ATGGCTTCACTTTCTCAACATCAACAC | biosynthesis |
| *DcAO* | AATGGTGGTAGTAGAGGCTGATGGAA | ATGGTTAGGGCTTGTGGTGTGTTG | recycling |
| *DcAPX* | AACAGTGAGCGAGGAGTACAAGGT | CAAGACGAAGCATAAGAGGAGCACAA | recycling |
| *DcMDHAR* | GGTGATAATGTTGGCGATGCTGTAATC | GCGATAGCCTTGTTCTCATCATCAGGAG | recycling |
| *DcDHAR* | CCAGAAGTGAAGTCCTATATGAAGACC | CAACCCTCTACGACATCCTCAGTTA | recycling |
| *DcGR* | ACACAGAAGAGTCACCTCAGGCTAT | CCACACCAACCGTCTCCAATCC | recycling |
| *Dctubulin* | GAGTGGAGTTACCTGCTGCCTTC | ATGTAGACGAGGGAACGGAATCAAG | _ |

**Table S2** Accession number of 12 related genes of ascorbic acid biosynthesis and recycling pathways in carrot taproot.

| Gene name | Accession number |
| --- | --- |
| *DcGGP* | KY347803 |
| *DcGME* | KY347804 |
| *DcGMP* | KY347805 |
| *DcGPP* | KY347806 |
| *DcPMI* | KY347807 |
| *DcAPX* | KR364573.1 |
| *DcPGI* | KY347808 |
| *DcAO* | KY347809 |
| *DcDHAR* | KY347810 |
| *DcGR* | KY347811 |
| *DcMDHAR* | KY347812 |
| *DcMIOX* | KY347813 |

**Figure S1**

Morphological changes in ‘Kurodagosun’ treated with a[CO_2_] and e[CO_2_].

Carrot samples at 38, 48, 58 and 68 days after sowing (DAS) were harvested under 400 and 3000 μmol·mol^−1^ CO_2_, respectively.


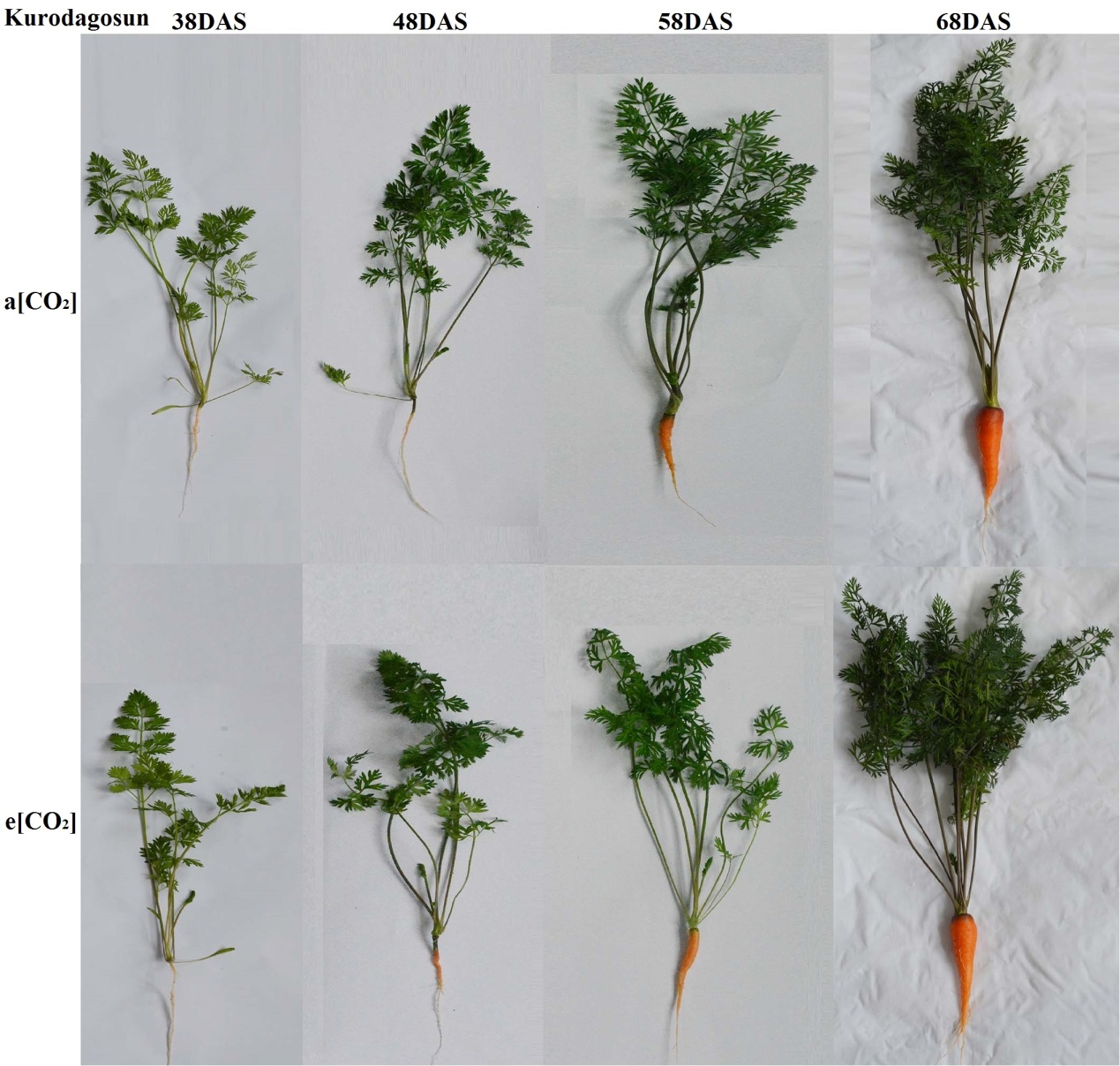


**Figure S2**

Morphological changes in ‘Deep purple’ treated with a[CO_2_] and e[CO_2_].

Carrot samples at 38, 48, 58 and 68 days after sowing (DAS) were harvested under 400 and 3000 μmol·mol^−1^ CO_2_.


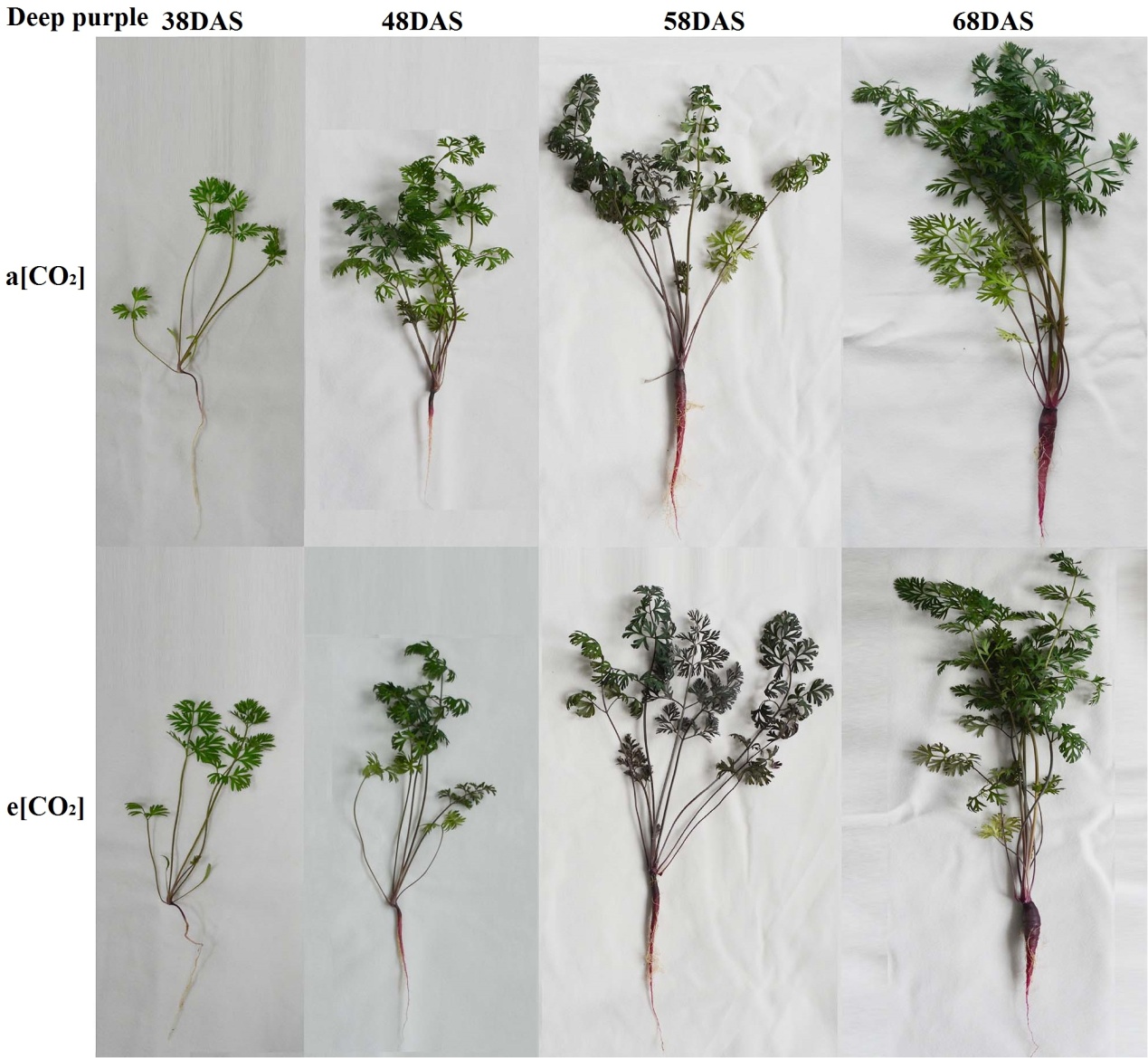

Supplement: Supplementary file 1 [file Data_Sheet_1.DOCX]
